# Length-dependent quantum interference and high thermoelectric response ferrocene-modified OPE wires

**DOI:** 10.1039/d6ra00084c

**Published:** 2026-03-06

**Authors:** Alaa A. Al-Jobory, Sameer Nawaf, Colin Lambert, Ali Ismael

**Affiliations:** a Physics Department, Lancaster University Lancaster LA1 4YB UK k.ismael@lancaster.ac.uk; b Department of Physics, College of Science, University of Anbar Ramadi Iraq; c Department of Physics, College of Education for Pure Science, Tikrit University Tikrit Iraq

## Abstract

We present a study of the length-dependent charge transport properties of a homologous series of oligo(phenylene–ethynylene) (OPE) molecular wires integrated with ferrocene units (Fe 1–Fe 5). Theoretical analysis reveals a coherent, length-dependent transport mechanism governed by quantum interference, with a distinct odd–even parity effect. Molecules with an odd number of ferrocene units exhibit a characteristic transmission dip within the HOMO–LUMO gap, a signature of destructive quantum interference (DQI), while even-numbered molecules show constructive quantum interference (CQI). This demonstrates that the interference behaviour is a holistic property of the full molecular length, not merely of the ferrocene core. The series exhibits efficient long-range tunneling, with a decay constant of *β* ≈ 1.1 nm^−1^ over lengths from 1.98 to 3.47 nm. Furthermore, these structures also possess high thermoelectric potential, with calculated Seebeck coefficients exceeding 250 µV K^−1^. The ability to switch between DQI and CQI states through molecular parity, combined with low conductance attenuation and high thermopower, establishes this ferrocene-OPE architecture as a highly promising platform for designing tuneable molecular electronic and energy conversion devices.

## Abstract 2

Chemical structure of ferrocene-modified OPE molecule with a variable number of repeating units (n = Fe 1 to Fe 5), and terminal end group thiol (SH).

## Graphical abstract

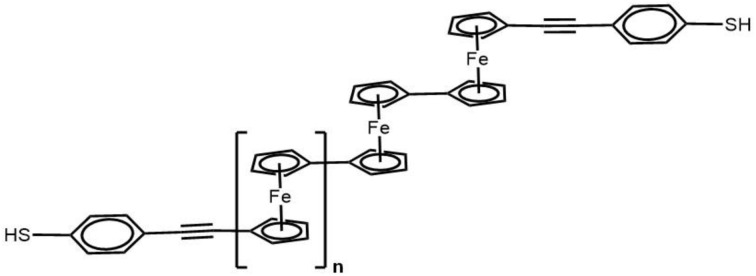

## 1 Introduction

Ferrocene, an archetypal organometallic compound discovered in 1951, features a distinctive sandwich structure in which an iron atom is symmetrically coordinated between two parallel cyclopentadienyl (C_5_H_5_) rings.^1–4^ This unique architecture confers exceptional stability, allowing it to persist in both aqueous and aerobic environments. Beyond its foundational role in organometallic chemistry, ferrocene has attracted significant interest in the field of molecular electronics.^5–10^ Its well-defined redox activity and distinctive electronic properties make it a promising candidate for constructing molecular junctions. A key aspect of its behaviour in such circuits is the potential manifestation of quantum interference (QI) effects.^10^ QI is a fundamental quantum phenomenon where constructive interference can enhance electron transport, or destructive interference can strongly suppress it, depending on the molecular structure and electronic pathways within the junction. The study of ferrocene thus provides a novel platform for exploring and harnessing these quantum effects for next-generation electronic devices. Constructive quantum interference (CQI) typically arises when electron waves traveling through different pathways such as the two branches of a *para*-connected, conjugated molecular structure exit the molecule in phase. Conversely, destructive quantum interference (DQI) occurs when electron waves become out of phase and destructively interfere, creating a pronounced antiresonance feature in the transmission spectrum that suppresses electron transport. This effect is a hallmark of quantum mechanics and is most famously observed in *meta*-connected aromatic systems, where the molecular topology inherently disrupts phase coherence. The unique value of ferrocene (bis(η^5^-cyclopentadienyl)iron) in this context lies in its mechanical flexibility and distinct electronic structure. The sandwich compound consists of two cyclopentadienyl rings that can rotate relative to each other with a low energy barrier, making the conformation specifically the dihedral angle between the rings' substituents a mechanically adjustable parameter. This rotational degree of freedom provides a direct handle for *in situ* control of quantum interference phenomena.^11–20^

As demonstrated by Camarasa-Gómez *et al.*, the electrical conductance of ferrocene derivatives can be substantially adjusted by mechanically manipulating the junction configuration. Their research elucidated that DQI in these systems stems from the precise hybridization between the iron atom's d-orbitals and the π-system of the organic ligands. By altering the molecular conformation for instance, by rotating the cyclopentadienyl rings, the electronic coupling between these orbitals is modulated. This tuning can either intensify the DQI by, pushing the antiresonance dip closer to the Fermi energy, or mitigate it to restore conductance, effectively turning the electron flow “off” and “on” through mechanical means. The ability to precisely control electron transport at the quantum level through a mechanical input paves the way for the development of ultra-sensitive molecular sensors, interferometric transistors, and non-linear circuit elements whose functionality is encoded directly into their structure and quantum properties. Ferrocene-based junctions, therefore, stand as a paradigm for the active and dynamic control of quantum effects in molecular-scale electronics.^21–25,45^

As demonstrated by Lu *et al.*,^26^ the energy separation between the highest occupied molecular orbital (HOMO) and the lowest unoccupied molecular orbital (LUMO) is a critical factor governing charge transport characteristics. Ferrocene, with its low-lying, readily accessible HOMO energy level, acts as a resonant gateway, enabling stronger electronic coupling with metal electrodes and promoting coherent tunnelling. This finding is strongly corroborated by the work of Sun *et al.*,^27^ who observed that inserting a ferrocene unit into insulating alkane chains resulted in a marked enhancement of electron transport. They ascribed this effect to the advantageous electronic interactions mediated by the organometallic ferrocene moiety, which creates a conductive pathway through an otherwise barrier-like chain. Beyond its favourable electronic properties, ferrocene's utility in molecular electronics and electrochemistry is multifaceted. Its stable and reversible redox behaviour provides a reliable switching mechanism and a well-defined energy state for electron transfer. These properties make ferrocene suitable for a wide range of applications, including molecular-scale charge transport, heterogeneous and homogeneous catalytic processes, and next-generation energy storage devices such as redox-flow batteries and electrochemical sensors.^28–36^

Theoretical investigations have significantly advanced our understanding of electron transport in molecular junctions incorporating ferrocene, a prototypical organometallic compound. Systematically examined the spin-filtering efficiency (SFE) in molecular junctions featuring ethynyl-terminated ferrocene derivatives. Their work demonstrated that the precise position of terminal group substitutions on the ferrocene core whether on the same or opposite cyclopentadienyl rings exerts a profound influence on the spin-polarized current–voltage (*I–V*) characteristics. This sensitivity arises because even minor modifications to the molecular configuration can drastically alter the electronic coupling between the ferrocene's frontier orbitals (notably the highest occupied molecular orbital, or HOMO) and the spin-polarised density of states in ferromagnetic electrodes. The inherent quantum nature of these ferrocene-based systems allows for the design of structures where QI effects are spin-selective, enabling exceptionally high spin-filtering performance.^37–40^

**Fig. 1:**
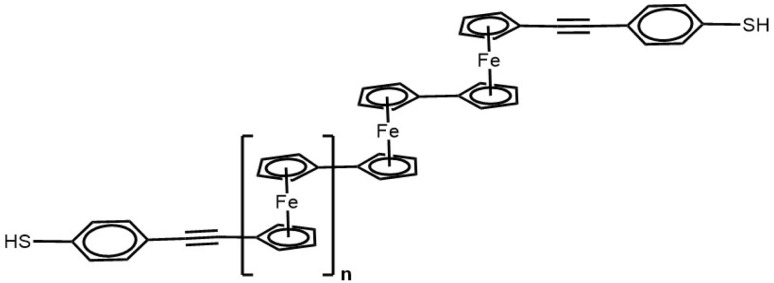
Chemical structure of ferrocene-modified OPE molecule with a variable number of repeating units (n = Fe 1 to Fe 5), and terminal end group thiol (SH).

In what follows, we presents a detailed theoretical investigation into the quantum thermoelectric transport properties of a series of ferrocene-modified oligo(phenylene ethynylene) (OPE) molecular junctions. The primary objective is to determine if quantum interference (QI) is constructive (CQI) or destructive (DQI) and to examine the evolution of electrical conductance and thermopower as a function of molecular length. Some of the OPE-derivatives under investigation have been synthesised^41^ and consist of a central ferrocene unit, functionalised with two terminal thiol groups for binding to gold electrodes. The molecules contain integrated OPE-based molecular backbones of varying lengths (denoted n = Fe 1 to Fe 5 OPE), as illustrated in Fig. 1. To describe their transport properties, we combine first-principles density functional theory (DFT) with non-equilibrium Green's function (NEGF) quantum transport simulations. This approach allows us to calculate their energy-dependent electron transmission spectra, *T*(*E*), which serve as the fundamental property for characterising conductance. The analysis of the transmission function near the Fermi energy (*E*_F_) provides direct signatures of QI effects, manifesting as either antiresonances (suppressed transmission indicative of DQI) or resonances (enhanced transmission indicative of CQI). In particular, this research addresses the question: how does the quantum interference signature originating from the ferrocene moiety evolve when the molecular bridge is extended from a short length (n = Fe 1) to a significantly longer one (n = Fe 5)? We hypothesise that increasing the length may not merely dampen the transmission uniformly, but could selectively suppress specific interfering pathways, potentially leading to a crossover between different QI regimes.

## 2 Methods

To calculate electron transport through the ferrocene core, we began by modelling the terminal SH–Au binding energetics and then relaxed each compound in the presence of fixed electrodes. Using the density functional code SIESTA,^42^ the optimum geometries of isolated ferrocene derivatives were obtained by relaxing the molecules until all forces on the atoms were less than 0.01 eV Å^−1^ (see Fig. S1). We used a double-zeta plus polarisation orbital basis set, norm-conserving pseudopotentials, the local density approximation (LDA) exchange–correlation functional, and to define the real space grid, an energy cutoff of 250 rydbergs. We also computed results using GGA and found that the resulting transmission functions were comparable^43,44^ with those obtained using LDA. To simulate the likely contact configuration during a break-junction experiment, we employed electrodes constructed from 5 layers of Au (111), each containing 30 gold atoms and further terminated with a pyramid of gold atoms. After relaxing each molecular junction in different orientations, we calculated the electrical conductance using the Gollum quantum transport code,^18^ (for more detail see the SI). Electron transport properties were evaluated in the linear-response (zero-bias) regime using the GOLLUM code. This approach utilises equilibrium Green's functions derived from the DFT-calculated Hamiltonian within the Landauer–Büttiker framework to determine the transmission coefficient rather than a self-consistent finite-bias NEGF implementation.

## 3 Results and discussion

The electronic properties of the ferrocene-modified OPE molecules (Fig. S1), were investigated through an analysis of their frontier molecular orbitals specifically, the highest occupied molecular orbital (HOMO) and the lowest unoccupied molecular orbital (LUMO). The energies and spatial distributions of these orbitals, are detailed in Fig. S2–S6. The optimal binding distance between the electrodes and the thiomethyl anchor groups were obtained by calculating their binding energies as a function of distance (Au–S), the covalent bond distance is found to be 2.4 Å, and the actual binding energy of approximately 1.9 eV, as illustrated in Fig. S7.

**Fig. 2:**
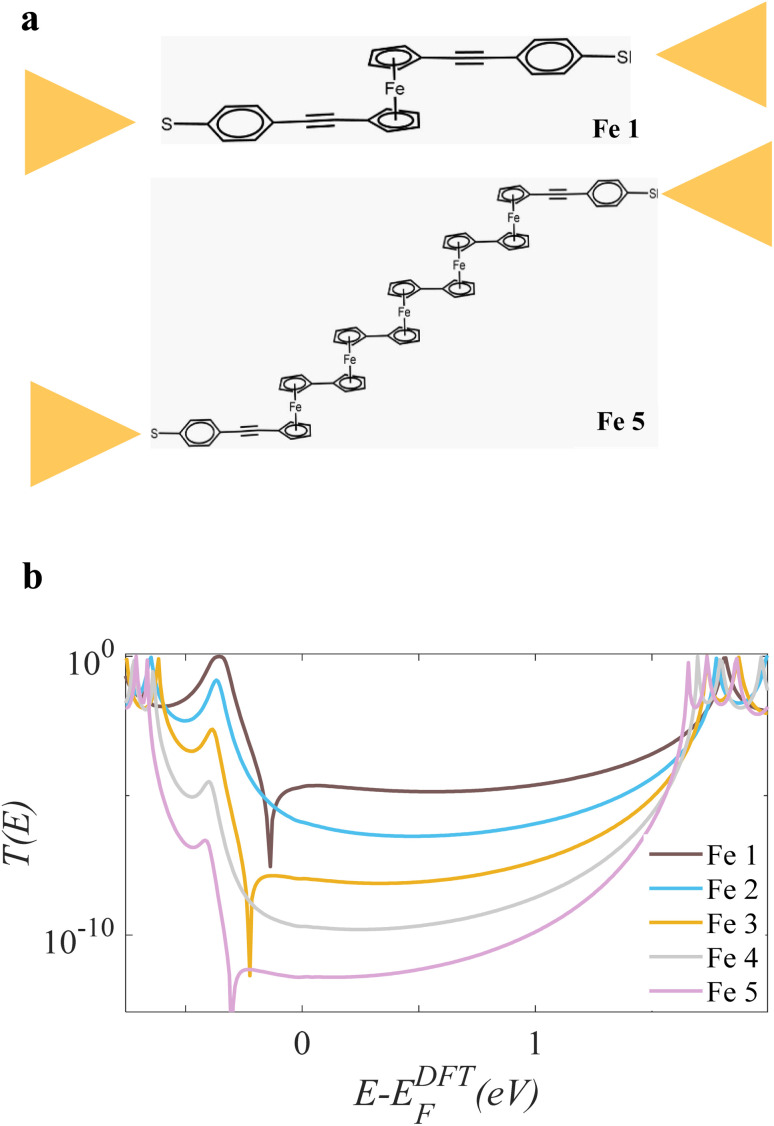
Structure and electron transmission of ferrocene-based molecular junctions. (a) Schematic of a single-molecule junction where an OPE-ferrocene derivative (illustrated with examples Fe 1 and Fe 5) bridges two gold electrodes. (b) Calculated zero-bias transmission spectra, *T*(*E*), for the five OPE-ferrocene molecules (Fe 1–Fe 5). The molecule-dependent shifts in the resonant peaks correspond to changes in the frontier orbital energies.

Fig. 2a below, shows the ferrocene-modified OPE molecules Fe 1 and Fe 5 connected to Au electrodes (Fe 2,Fe 3 and Fe 4 are not shown here; for more details, see Fig. S8). These molecules consist of a rigid oligo(phenylene ethynylene) (OPE) backbone, which serves as a molecular wire, functionalized with a redox-active ferrocene moiety. Given the presence of a transition metal centre (Fe) in the ferrocene-modified derivatives, spin-polarised density functional theory (DFT) calculations were necessary to accurately model their electronic structure. The corresponding transmission functions for both spin-up and spin-down channels are presented in Fig. S9 (SI). The results reveal an absence of spin polarisation, as evidenced by the identical transmission spectra for the two spin channels across all five OPE derivatives.

Fig. 2b presents the corresponding quantum transport characteristics for the five derivatives, revealing a mixed regime of quantum interference behaviour. This is manifested in the calculated transmission functions, *T*(*E*), which exhibit two distinct profiles near the HOMO (Highest Occupied Molecular Orbital) resonance energy: DQI is characterised by the presence of a pronounced dip in the transmission function.

The zero-bias transmission spectra in Fig. 2b reveal that the quantum interference (QI) in these junctions is molecule-dependent. A distinct even–odd effect is observed: junctions with an odd number of ferrocene units (Fe 1, Fe 3, Fe 5) exhibit a characteristic dip near the HOMO resonance, a signature of DQI. In contrast, junctions with an even number of units (Fe 2, Fe 4) lack this dip, displaying a smoother profile typical of CQI. The relationship between orbital symmetry and quantum interference (QI) was evaluated by examining the product of HOMO and LUMO coefficients at the connection sites. A positive product indicates constructive quantum interference (CQI), enhancing electron transmission, whereas a negative product signifies destructive quantum interference (DQI), which suppresses conductance. This symmetry-based analysis provides a clear rationale for the transmission features observed across the different molecular configurations. As discussed in Section 1.2 of the SI, this odd–even effect is consistent with the alternating symmetries of the HOMO and LUMO molecular orbitals.^18^

**Fig. 3:**
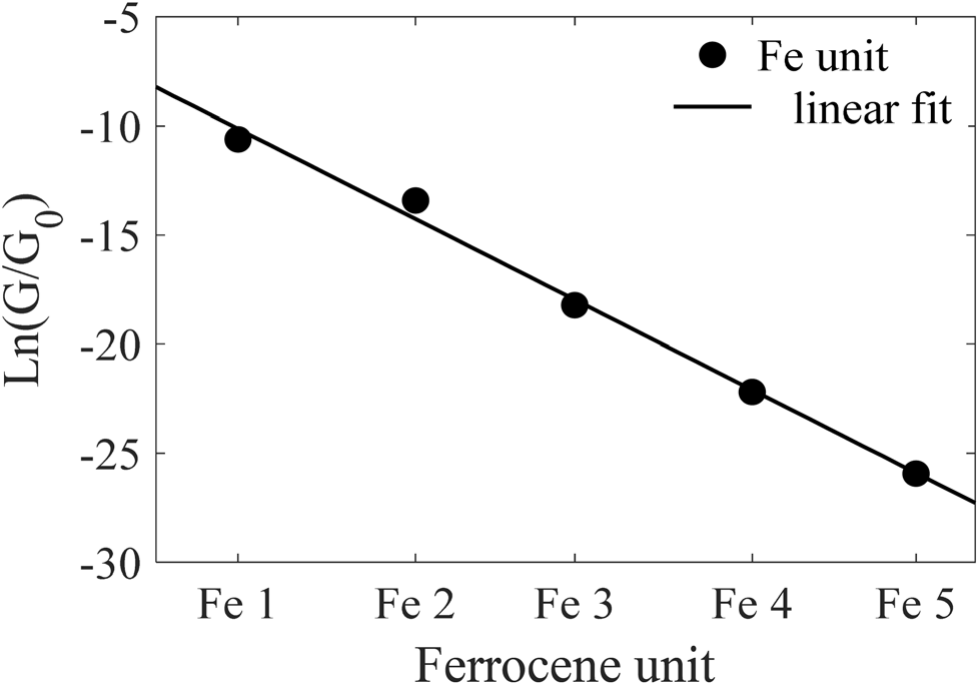
Tunnelling decay factor as a function of the molecular length for the five OPE-ferrocene molecules (Fe 1–Fe 5).

To quantitatively characterize the charge transport mechanism across the ferrocene-based molecular junctions (Fe 1–Fe 5), we calculated the tunneling decay factor (*β*) using the established relationship for non-resonant tunneling:

1 ln(*G*(*L*)) = ln(*G*(0)) − *βL*

where *G*(*L*) is the simulated molecular conductance, *L* is the effective tunneling length defined as the distance between the sulfur anchoring atoms and *G*(0) represents the contact conductance extrapolated to zero length. The measured molecular lengths for the series are 1.98 nm (Fe 1), 2.37 nm (Fe 2), 2.72 nm (Fe 3), 3.11 nm (Fe 4), and 3.47 nm (Fe 5). The observed linear relationship between ln(*G*) and *L* unambiguously confirms that electron transport occurs *via* a direct through-bond tunneling mechanism (see Fig. 3). This is a direct consequence of the significant energy barrier presented by the ferrocene units, whose frontier molecular orbitals are energetically misaligned with the electrodes' Fermi level.

The thermoelectric performance of a molecular junction is captured by the Seebeck coefficient (*S*), which quantifies the voltage generated in response to a temperature difference across the junction. At the molecular scale, the Seebeck coefficient is governed by the electronic transmission spectrum, *T*(*E*), which describes the probability of an electron at energy *E* tunneling through the molecule. The relationship is given by a derivative of the transmission function at the Fermi energy (*E*_F_) of the electrodes:2
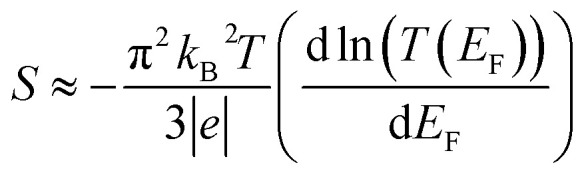


This equation reveals that the Seebeck coefficient is proportional to the logarithmic derivative of the transmission function, d ln *T*(*E*)/d*E*, evaluated at *E*_F_. Physically, this derivative reflects the slope of the transmission function around the Fermi level. A positive Seebeck coefficient (p-type behaviour) arises when the slope <0 at *E*_F_.

**Fig. 4:**
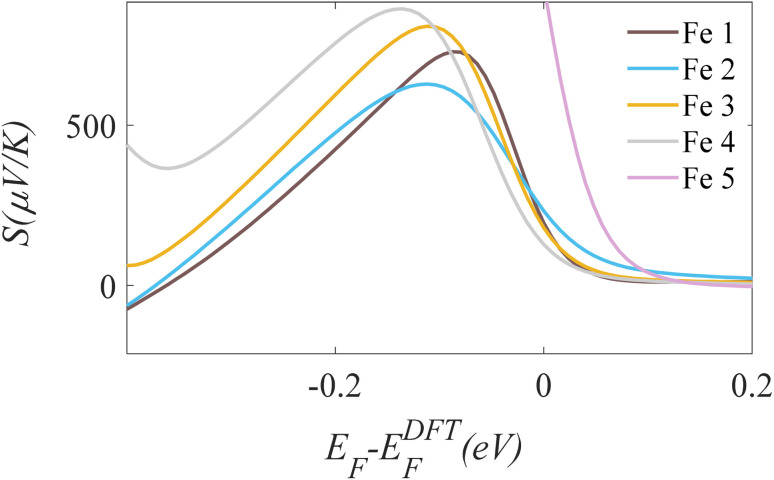
Calculated Seebeck coefficient as a function of electron energy for the OPE-ferrocene series (Fe 1–Fe 5).

This is typically occurring when the is close to the HOMO, creating a steep “downward slope” in *T*(*E*) at the Fermi energy. Conversely, a negative Seebeck coefficient (n-type behaviour) occurs when the Fermi energy lies just below the LUMO. In this study, the calculated Seebeck coefficients for the studied molecules are all positive at the DFT-predicted Fermi energy (*E*_F_ − *E*^DFT^_F_ = 0 eV), as shown in Fig. 4.

This HOMO-dominated transport is consistent with the electronic structure of ferrocene, whose ionization potential places its occupied states near typical electrode Fermi levels. The positive Seebeck coefficient thus confirms the p-type character of these molecular junctions and shows that thermoelectric transport is governed by hole conduction *via* the HOMO-dominated pathway. While standard DFT tends to underestimate fundamental energy gaps due to self-interaction errors and the lack of derivative discontinuity, these effects are systematic across our consistent computational framework. Because our focus remains on relative trends induced by structural functionalization and electrode coupling rather than absolute orbital energies, the comparative conclusions regarding electronic transport and level alignment remain qualitatively robust. The observed interference effects originate from quantum phase coherence between multiple electron transmission pathways through the π-conjugated backbone of the molecule. Constructive interference occurs when electron waves propagating along different conjugation paths accumulate phases that are in-phase at the electrode–molecule interfaces, leading to enhanced transmission near the Fermi level, whereas destructive interference arises when these pathways acquire a phase difference close to π, resulting in transmission suppression and anti-resonances. The even–odd effect is a direct consequence of the parity of the molecular backbone (or the number of repeat units), which determines the symmetry of the frontier molecular orbitals and the relative phase of the wavefunctions at the anchoring sites. In even systems, the electrode coupling occurs through orbitals with opposite phase at the two contacts, favouring destructive interference, while odd systems preserve phase continuity and therefore support constructive interference.

## 4 Conclusions

The systematic investigation of charge transport through the ferrocene-based molecular wire series, Fe 1–Fe 5, reveals that their electrical properties are governed by a coherent interplay of molecular length and quantum interference. We observed a clear parity rule: molecules with an odd number of ferrocene units exhibit a pronounced conductance dip within the HOMO–LUMO gap, indicative of destructive quantum interference. Conversely, even-numbered molecules display constructive interference, evident in a smooth transmission function across the gap. This clear odd–even effect demonstrates that quantum interference is not an intrinsic feature of the isolated ferrocene unit but a holistic, emergent property dictated by the entire molecular conjugation pathway.

The efficient long-range transport in these wires is further confirmed by the relatively low tunneling decay constant (*β* ∼ 1.1 nm^−1^) obtained across the length series (1.98–3.47 nm). Furthermore, the molecular architecture also exhibits exceptional potential for energy conversion, with calculated Seebeck coefficients surpassing 250 µV K^−1^ near the Fermi level. The synergistic combination of tuneable quantum interference, low conductance decay, and high thermopower establishes this ferrocene-OPE platform as a highly versatile foundation for next-generation molecular-scale devices, including quantum-interference transistors and high-performance molecular thermoelectrics.

## Author contributions

A. K. I. originally conceived the concept; calculations were carried out by A. A. and S. N. All authors have given approval to the final version of the manuscript. All authors provided essential contributions to interpreting the data reported in this manuscript. A. K. I. coordinated the writing of the manuscript with input from A. A. and C. L.

## Conflicts of interest

There are no conflicts to declare.

## Supplementary Material

RA-016-D6RA00084C-s001

## Data Availability

In this work, we use the following codes: (1) Siesta code used to predict the Hamiltonian of each system used in this study, which is located in https://gitlab.com/siesta-project/siesta/-/releases. (2) GOLLUM software is used to find the website's transmission coefficient, as well to calculate Conductance, Seebeck and all other parameters: https://www.gollumcode.com/. Supplementary information: provides the optimized DFT geometries, interfacial binding energies, and quantum transport properties specifically transmission and Seebeck coefficients for the studied molecular junctions. See DOI: https://doi.org/10.1039/d6ra00084c.

## References

[cit1] Astruc D. (2017). Why is Ferrocene so Exceptional?. Eur. J. Inorg. Chem..

[cit2] Wilkinson L. A., Bennett T. L., Grace I. M., Hamill J., Wang X., Au-Yong S., Ismael A., Jarvis S. P., Hou S., Albrecht T. (2022). Assembly, structure and thermoelectric properties of 1, 1′-dialkynylferrocene ‘hinges’. Chem. Sci..

[cit3] Snegur L. V. (2022). Modern Trends in Bio-Organometallic Ferrocene Chemistry. Inorganics.

[cit4] Bennett T. L., Alshammari M., Au-Yong S., Almutlg A., Wang X., Wilkinson L. A., Albrecht T., Jarvis S. P., Cohen L. F., Ismael A. (2022). Multi-component self-assembled molecular-electronic films: towards new high-performance thermoelectric systems. Chem. Sci..

[cit5] van Staveren D. R., Metzler-Nolte N. (2004). Bioorganometallic Chemistry of Ferrocene. Chem. Rev..

[cit6] Astruc D. (2023). From sandwich complexes to dendrimers: journey toward applications to sensing, molecular electronics, materials science, and biomedicine. Chem. Commun..

[cit7] Ismael A. K., Lambert C. J. (2020). Molecular-scale thermoelectricity: a worst-case scenario. Nanoscale Horiz..

[cit8] Larik F. A., Saeed A., Fattah T. A., Muqadar U., Channar P. A. (2017). Recent advances in the synthesis, biological activities and various applications of ferrocene derivatives. Appl. Organomet. Chem..

[cit9] Werner H. (2012). At Least 60 Years of Ferrocene: The Discovery and Rediscovery of the Sandwich Complexes. Angew. Chem., Int. Ed..

[cit10] Siemeling U. (2012). Singlet Carbenes Derived from Ferrocene and Closely Related Sandwich Complexes. Eur. J. Inorg. Chem..

[cit11] Al-Jobory A. A., Noori M. D. (2020). Thermoelectric properties of metallocene derivative single-molecule junctions. J. Electron. Mater..

[cit12] Jia C., Grace I. M., Wang P., Almeshal A., Huang Z., Wang Y., Chen P., Wang L., Zhou J., Feng Z., Zhao Z., Huang Y., Lambert C. J., Duan X. (2020). Redox Control of Charge Transport in Vertical Ferrocene Molecular Tunnel Junctions. Chem.

[cit13] Ye J., Al-Jobory A., Zhang Q.-C., Cao W., Alshehab A., Qu K., Alotaibi T., Chen H., Liu J., Ismael A. K. (2022). Highly insulating alkane rings with destructive σ-interference. Sci. China: Chem..

[cit14] Al-Jobory A. A., Ismael A. K. (2023). Controlling quantum interference in tetraphenyl-aza-BODIPYs. Curr. Appl Phys..

[cit15] Alanazi B., Alajmi A., Aljobory A., Lambert C., Ismael A. (2024). Tuning quantum interference through molecular junctions formed from cross-linked OPE-3 dimers. J. Mater. Chem. C.

[cit16] Chen F., Liang Q.-M., Lin L.-X., Zhang Q.-C., Yang Y. (2023). Recent progress in tuning charge transport in single-molecule junctions by substituents. J. Mater. Chem. C.

[cit17] Wang X., Ismael A., Ning S., Althobaiti H., Al-Jobory A., Girovsky J., Astier H. P., O'Driscoll L. J., Bryce M. R., Lambert C. J. (2022). Electrostatic Fermi level tuning in large-scale self-assembled monolayers
of oligo (phenylene–ethynylene) derivatives. Nanoscale Horiz..

[cit18] Lambert C. J., Liu S. X. (2018). A magic ratio rule for beginners: a chemist's guide to quantum interference in molecules. Chem.–Eur. J..

[cit19] Solomon G. C., Bergfield J. P., Stafford C. A., Ratner M. A. (2011). When “small” terms matter: Coupled interference features in the transport properties of cross-conjugated molecules. Beilstein J. Nanotechnol..

[cit20] Alresheedi K., Alajmi A., Alrehaili A., Al-Jobory A., Lambert C., Ismael A. (2025). Orientational control of quantum interference in ferrocene single-molecule junctions. Mater. Chem. Front..

[cit21] Camarasa-Gómez M., Hernangómez-Pérez D., Inkpen M. S., Lovat G., Fung E. D., Roy X., Venkataraman L., Evers F. (2020). Mechanically Tunable Quantum Interference in Ferrocene-Based Single-Molecule Junctions. Nano Lett..

[cit22] Naher M. , Structure-Property Relationships in Molecular Electronics, 2021

[cit23] Ismael A., Wang X., Al-Jobory A., Ning S., Alotaibi T., Alanazi B., Althobaiti H., Wang J., Wei N., Ford C. J. (2024). Tuning the electrical conductance of oligo (phenylene-ethynylene) derivatives-PbS quantum-dot bilayers. J. Mater. Chem. C.

[cit24] Zhao X., Kastlunger G., Stadler R. (2017). Quantum interference in coherent tunneling through branched molecular junctions containing ferrocene centers. Phys. Rev. B.

[cit25] Alshehab A., Ismael A. K. (2023). Impact of the terminal end-group on the electrical conductance in alkane linear chains. RSC Adv..

[cit26] Wang X., Alajmi A., Wei Z., Alzanbaqi M., Wei N., Lambert C., Ismael A. (2024). Enhancing the Pressure-Sensitive Electrical Conductance of Self-Assembled Monolayers. ACS Appl. Mater. Interfaces.

[cit27] Lu Q., Yao C., Wang X., Wang F. (2012). Enhancing Molecular Conductance of Oligo(p-phenylene ethynylene)s by Incorporating Ferrocene into Their Backbones. J. Phys. Chem. C.

[cit28] Sun Y.-Y., Peng Z.-L., Hou R., Liang J.-H., Zheng J.-F., Zhou X.-Y., Zhou X.-S., Jin S., Niu Z.-J., Mao B.-W. (2014). Enhancing electron transport in molecular wires by insertion of a ferrocene center. Phys. Chem. Chem. Phys..

[cit29] Zhang L., Zhao Y., Kong W., Zhang H., Zang L., Zhao M., Zhang J., Kong R. M., Zhang E. S., Qu F. (2025). Functional Metallocenes as Cofactors Promote the Catalytic Performance of Mimetic Enzymes. Small.

[cit30] Takashima H., Inagaki Y., Momma H., Kwon E., Yamaguchi K., Setaka W. (2018). Ferrocene-diyl bridged macrocages: steric effects of the cage on the redox properties of ferrocene moiety. Organometallics.

[cit31] Jeong H., Jang Y., Kim D., Hwang W.-T., Kim J.-W., Lee T. (2016). An in-depth study of redox-induced conformational changes in charge transport characteristics of a ferrocene-alkanethiolate molecular electronic junction: Temperature-dependent transition voltage spectroscopy analysis. J. Phys. Chem. C.

[cit32] Alshammari M., Alhassan S., Alshammari K., Alotaibi T., Alshammari A. H., Alotibi S., Taha T. A. M., Ismael A. (2023). Hydrogen catalytic performance of hybrid Fe3O4/FeS2/g-C3N4 nanocomposite structures. Diamond Relat. Mater..

[cit33] Cai Y., Gao Y., Luo Q., Li M., Zhang J., Tian H., Zhu W. H. (2016). Ferrocene-grafted photochromic triads based on a sterically hindered ethene bridge: Redox-switchable fluorescence and gated photochromism. Adv. Opt. Mater..

[cit34] Taha T. A. M., Alanazi S. S., El-Nasser K. S., Alshammari A. H., Ismael A. (2024). Structure–property relationships in PVDF/SrTiO3/CNT nanocomposites for optoelectronic and solar cell applications. Polymers.

[cit35] Ismael A. K. (2023). 20-State Molecular Switch in a Li@ C60 Complex. ACS Omega.

[cit36] Yokota Y., Mino Y., Kanai Y., Utsunomiya T., Imanishi A., Fukui K.-i. (2015). Electronic-state changes of ferrocene-terminated self-assembled monolayers induced by molecularly thin ionic liquid layers: A combined atomic force microscopy, X-ray photoelectron spectroscopy, and ultraviolet photoelectron spectroscopy study. J. Phys. Chem. C.

[cit37] Hou J.-L., Luo W., Guo Y., Zhang P., Yang S., Zhu Q.-Y., Dai J. (2017). Titanium oxo cluster with six peripheral ferrocene units and its photocurrent response properties for saccharides. Inorg. Chem..

[cit38] Yuan S., Wang S., Kong Z., Xu Z., Yang L., Wang D., Ling Q., Wang Y. (2018). Theoretical Studies of the Spin-Dependent Electronic Transport Properties in Ethynyl-Terminated Ferrocene Molecular Junctions. Micromachines.

[cit39] Gryaznova T. P., Katsyuba S. A., Milyukov V. A., Sinyashin O. G. (2010). DFT study of substitution effect on the geometry, IR spectra, spin state and energetic stability of the ferrocenes and their pentaphospholyl analogues. J. Organomet. Chem..

[cit40] Ismael A., Al-Jobory A., Wang X., Alshehab A., Almutlg A., Alshammari M., Grace I., Benett T. L., Wilkinson L. A., Robinson B. J. (2020). Molecular-scale thermoelectricity: as simple as ‘ABC’. Nanoscale Adv..

[cit41] Ismael A. K., Mohaymen Taha T. A., Al-Jobory A. (2024). Three distinct conductance states in polycyclic aromatic hydrocarbon derivatives. R. Soc. Open Sci..

[cit42] Chen C.-P., Luo W.-R., Chen C.-N., Wu S.-M., Hsieh S., Chiang C.-M., Dong T.-Y. (2013). Redox-active π-conjugated organometallic monolayers: Pronounced Coulomb blockade characteristic at room temperature. Langmuir.

[cit43] Soler J. M., Artacho E., Gale J. D., García A., Junquera J., Ordejón P., Sánchez-Portal D. (2002). The SIESTA method for ab initio order-N materials simulation. J. Phys.: Condens. Matter.

[cit44] Ismael A. K., Al-Jobory A., Grace I., Lambert C. J. (2017). Discriminating single-molecule sensing by crown-ether-based molecular junctions. J. Chem. Phys..

[cit45] Ismael A. K., Grace I., Lambert C. J. (2015). Increasing the thermopower of crown-ether-bridged anthraquinones. Nanoscale.

