# Supplementary material for: Length-dependent quantum interference and high thermoelectric response ferrocene-modified OPE wires
Source: RSC Adv. 2026 Mar 6;16(14):12730–6. doi: 10.1039/d6ra00084c (PMC12965210; doi:10.1039/d6ra00084c)
Supplement: RA-016-D6RA00084C-s001 [file RA-016-D6RA00084C-s001.pdf]

Supplementary Information (SI) for RSC Advances.
This journal is © The Royal Society of Chemistry 2026

Supporting Information

Length-Dependent Quantum Interference and High Thermoelectric Response Ferrocene-Modified OPE Wires

Alaa A. Al-Jobory[a,b], Sameer Nawaf[b], Colin Lambert,[a] and Ali Ismael[a,c*]

a. Physics Department, Lancaster University, Lancaster, LA1 4YB, UK.
b. Department of Physics, College of Science, University of Anbar, Ramadi, Iraq.
c. Department of Physics, College of Education for Pure Science, Tikrit University, Tikrit, Iraq.

Contents

1.1 Optimised DFT structures of isolated molecules 4
1.2 Binding energy 12
1.3 Transmission coefficient $T(E)$ 18
1.4 Seebeck coefficient 24
2. Referencing 26

1.1- Optimised DFT structures of isolated molecules

Density functional theory (DFT) [1], [2], [3] calculations were performed using the SIESTA code to obtain the optimised geometries of all isolated macrocyclic molecules presented in this study. The geometries were relaxed until the atomic forces were reduced below $0.01\ eV/A^o$. A double-zeta plus polarization basis set (DZP) with a 250 Rydberg energy cut-off was *employed*, and the generalized gradient approximation (GGA) was used as the exchange-correlation functional. These optimised structures serve as the basis for further electronic and transport property calculations. The DFT-optimised geometries of the ferrocene-modified OPE molecules are presented in **Figure S 1**, which were analysed to identify specific molecular length configurations associated with high electrical conductance (constructive quantum interference, CQI) and low conductance (destructive quantum interference, DQI).

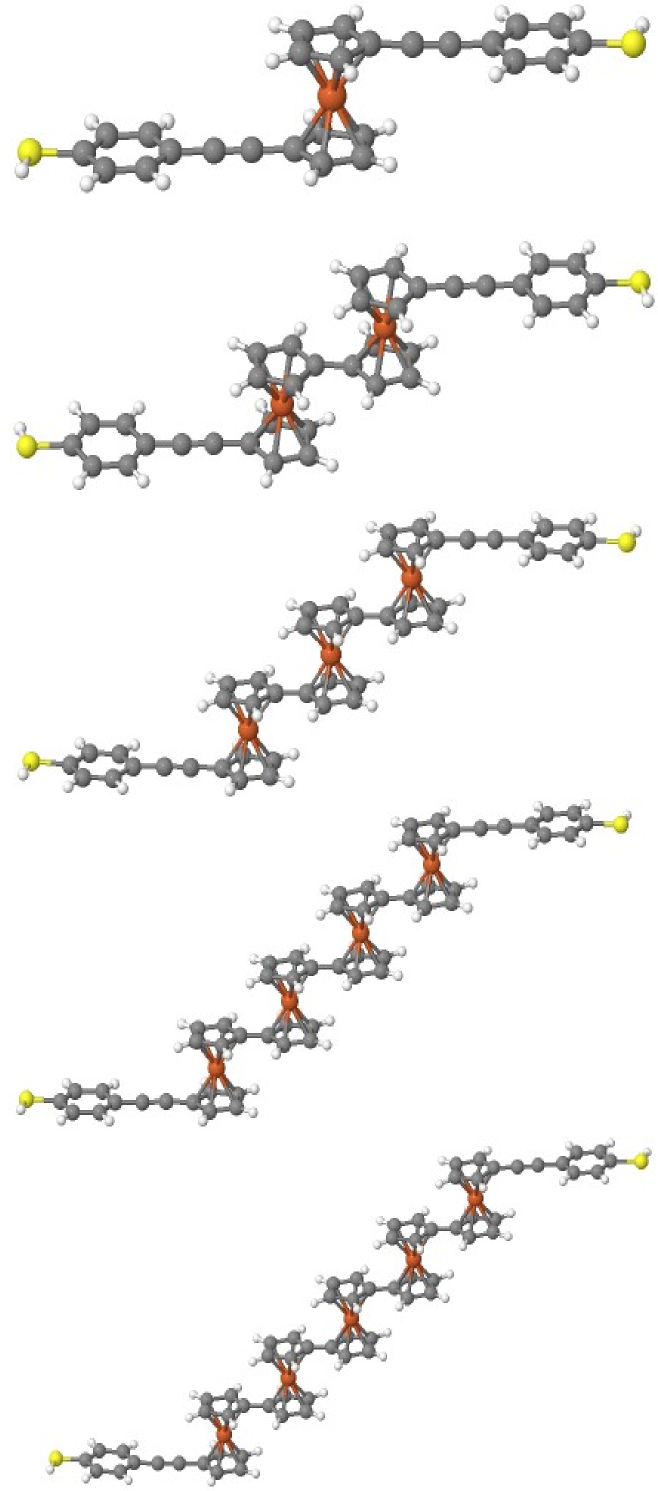

Figure S1: DFT-optimised molecular structures of ferrocene derivatives (**Fe 1–Fe 5**). Computationally relaxed geometries reveal the intrinsic conformational flexibility and three-dimensional topology of the isolated molecules prior to junction formation and quantum transport simulation.

1.2- Frontier orbitals of the molecules

To gain insight into the electronic properties of the structures depicted in Figure (S. 1), we used DFT to investigate the molecular orbitals of every gas-phase derivative. The frontier orbitals of the ferrocene-modified OPE molecules **Fe 1– Fe 5** (see Figure (S. 1)), are shown in Figure (S. 2) - Figure (S. 6). Their highest-occupied molecular orbitals (HOMO) and lowest unoccupied orbitals (LUMO), and additional orbitals were calculated along with their energies. The yellow and green colours represent the positive and negative orbital amplitudes. These orbitals are of interest, because the relative symmetry of the HOMO and LUMO can be used as a qualitative predictor of the occurrence of CQI or DQI. For example, Fig (S2) shows that the HOMO amplitude on the left end of the molecule is positive and on the right end is negative, so the product of these HOMO amplitudes is negative. Similarly, the LUMO amplitude on the left end of the molecule is positive and on the right end is negative, so the product of these LUMO amplitudes is negative. If the HOMO product has the same sign as the LUMO product (as in this case), then DQI is expected. On the other hand, if the HOMO product has a different sign than the LUMO product (as in Fig. S3), then CQI is expected. Inspection of the HOMO and LUMO orbitals below reveals that DQI is expected for molecules with an odd number of ferrocenes and CQI is expected for molecules with an even number of ferrocenes.

Fe 1:

$E_F = -\ 2.98\ eV$

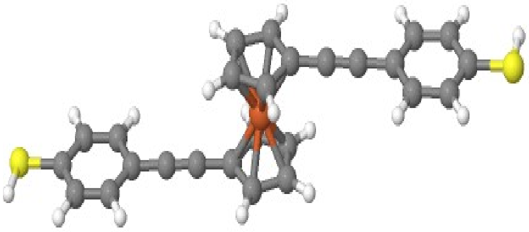

HOMO= -4.12 eV

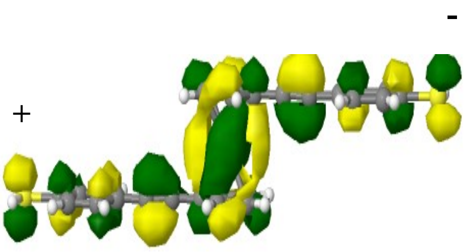

LUMO= -1.92 eV

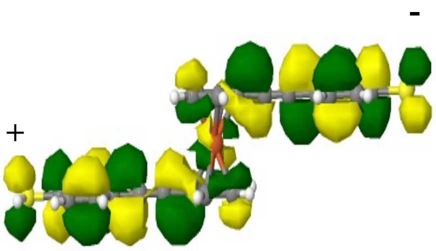

HOMO-1 = -4.32 eV

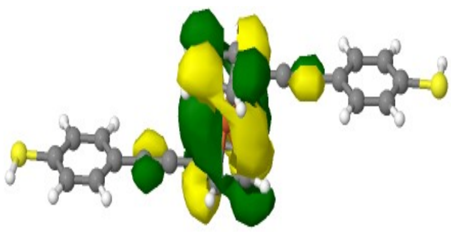

LUMO+1= -1.56 eV

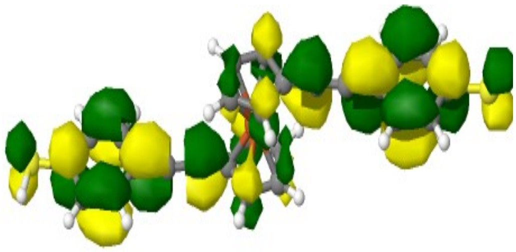

Figure (S2). Frontier molecular orbital landscape of ferrocene derivative **Fe1**. Computed electronic structures showing the highest occupied molecular orbital (HOMO), lowest unoccupied molecular orbital (LUMO), and their energy levels (HOMO-1 and LUMO+1), illustrating the spatial distribution and energetic relationships of key orbitals involved in charge transport. Here the HOMO and LUMO products are both negative, indicating DQI.

Fe 2:

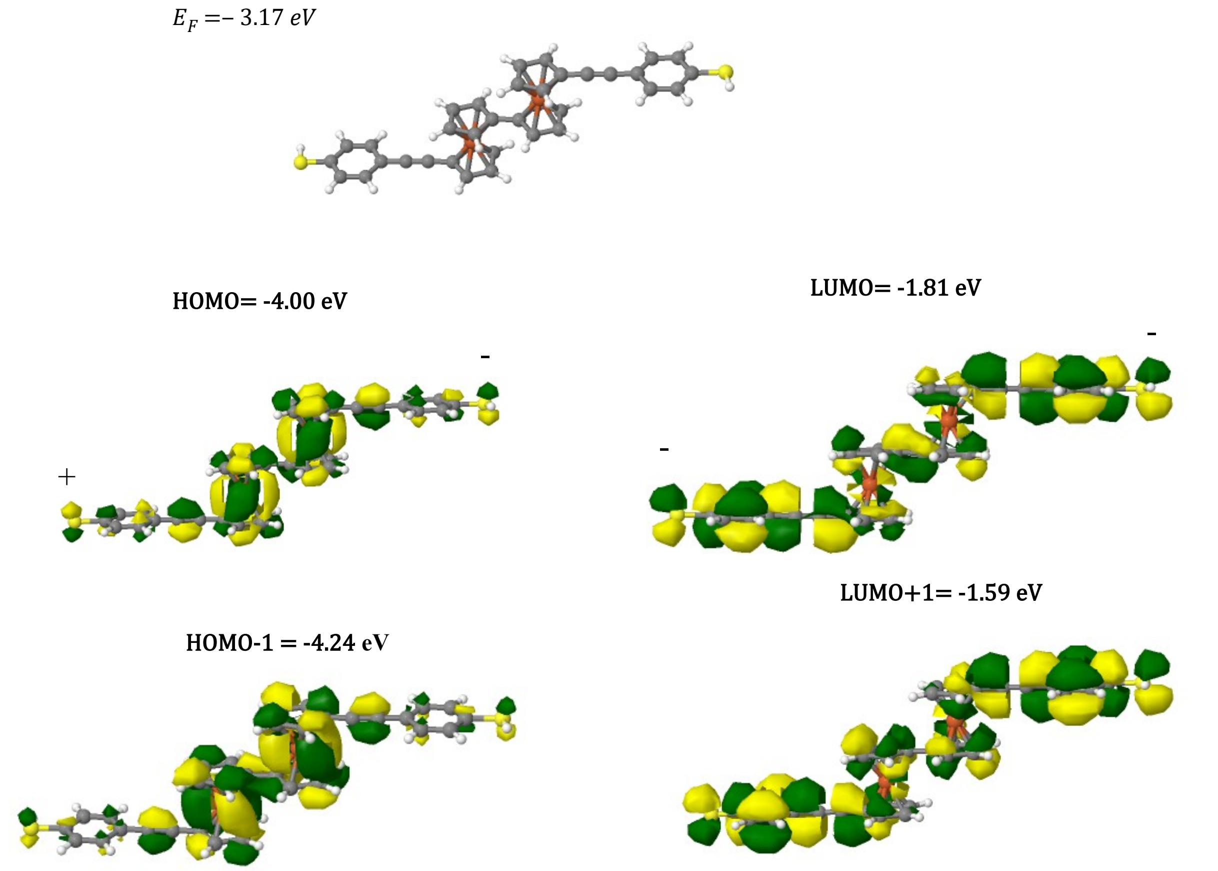

Figure (S3). Frontier molecular orbital landscape of ferrocene derivative **Fe 2**. Computed electronic structures showing the highest occupied molecular orbital (HOMO), lowest unoccupied molecular orbital (LUMO), and their energy levels (HOMO-1 and LUMO+1), illustrating the spatial distribution and energetic relationships of key orbitals involved in charge transport. Here the HOMO product is negative and the LUMO product is positive indicating CQI.

Fe 3:

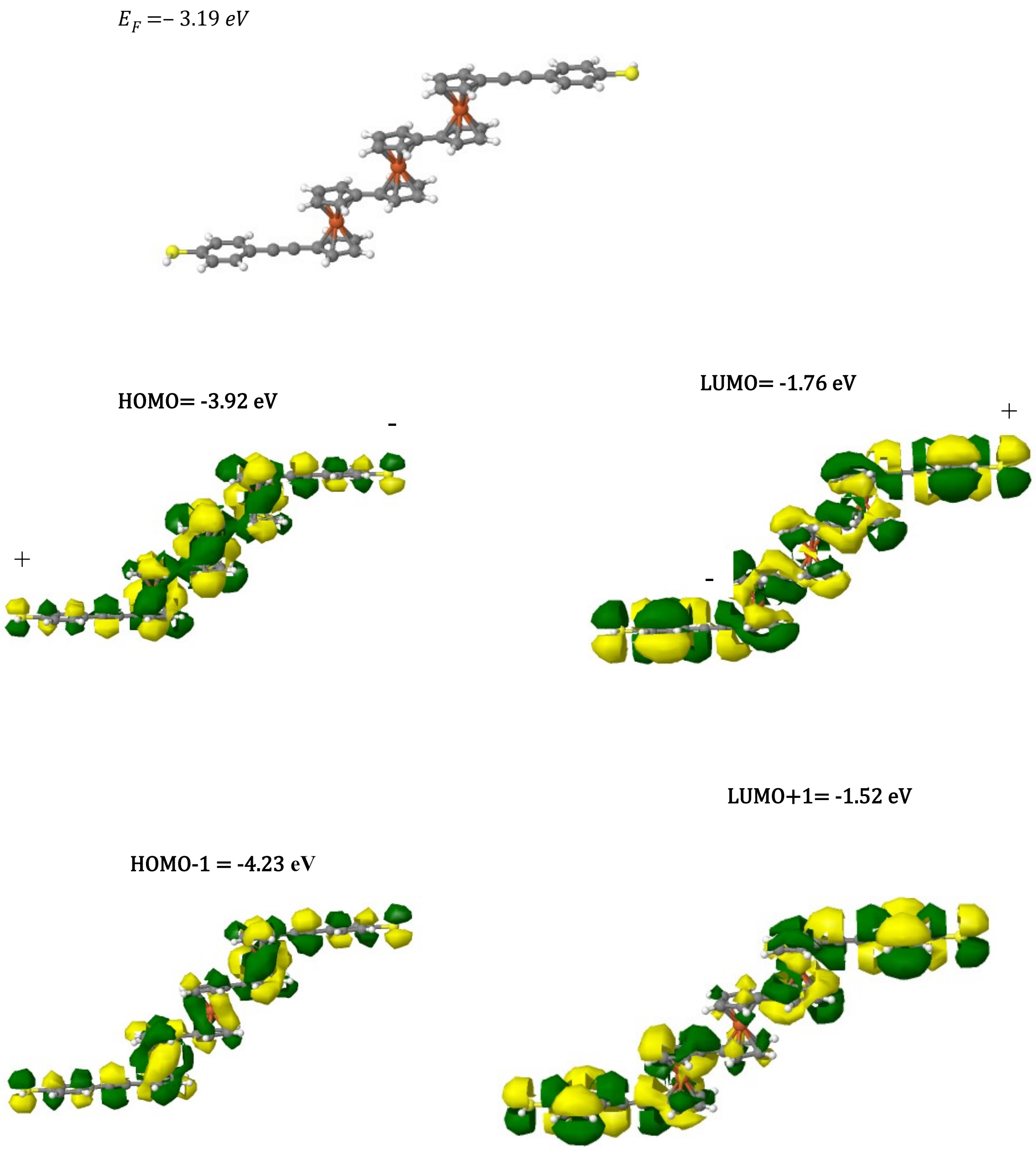

Figure (S4). Frontier molecular orbital landscape of ferrocene derivative **Fe 3**. Computed electronic structures showing the highest occupied molecular orbital (HOMO), lowest unoccupied molecular orbital (LUMO), and their energy levels (HOMO-1 and LUMO+1), illustrating the spatial distribution and energetic relationships of key orbitals involved in charge transport. Here the HOMO and LUMO products are both negative, indicating DQI.

Fe 4:

$E_F = -3.18\ eV$

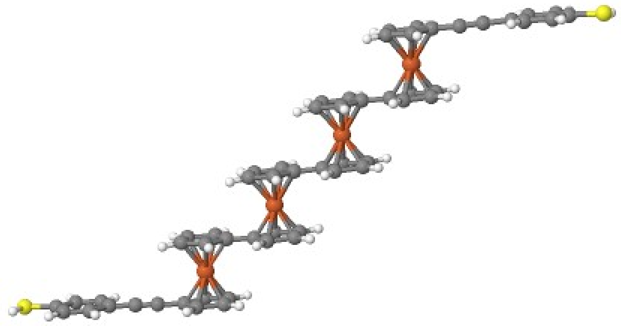

HOMO= -3.87 eV

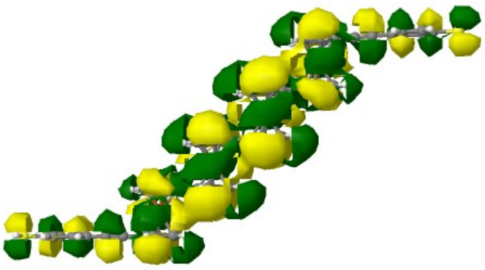

LUMO= -1.74 eV

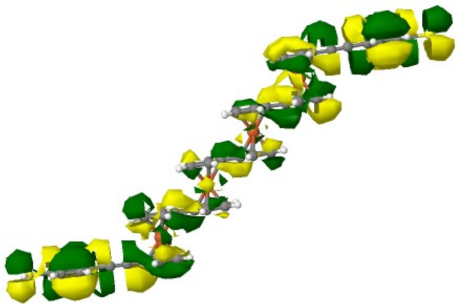

HOMO-1 = -4.16 eV

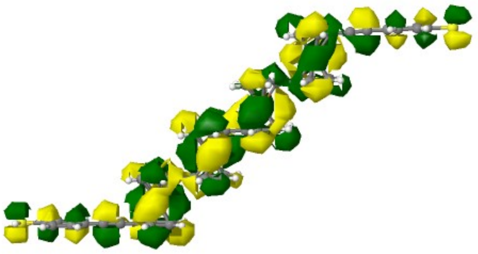

LUMO+1= -1.53 eV

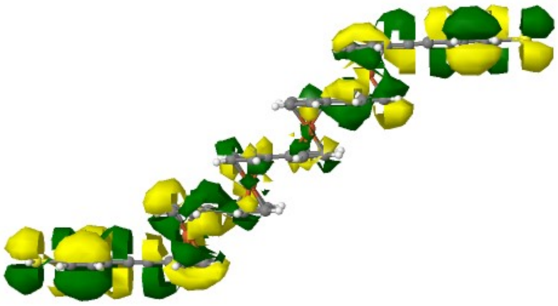

Figure (S5). Frontier molecular orbital landscape of ferrocene derivative **Fe 4**. Computed electronic structures showing the highest occupied molecular orbital (HOMO), lowest unoccupied molecular orbital (LUMO), and their energy levels (HOMO-1 and LUMO+1), illustrating the spatial distribution and energetic relationships of key orbitals involved in charge transport. Here the HOMO product is negative and the LUMO product is positive indicating CQI.

Fe 5:

$E_F = -3.17\ eV$

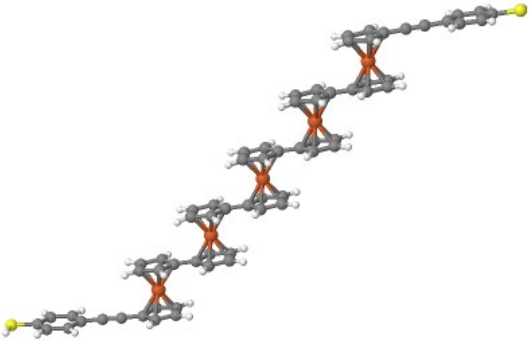

HOMO= -3.83 eV

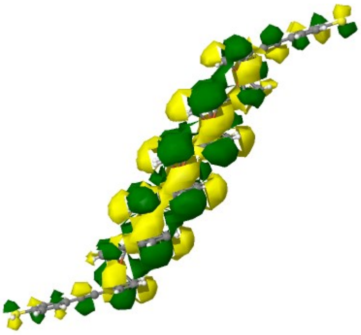

LUMO= -1.73 eV

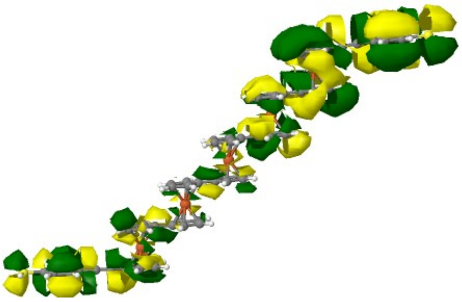

HOMO-1 = -4.16 eV

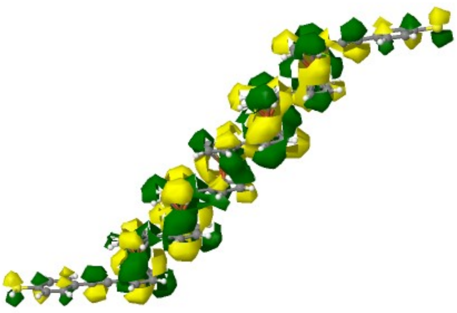

LUMO+1= -1.52 eV

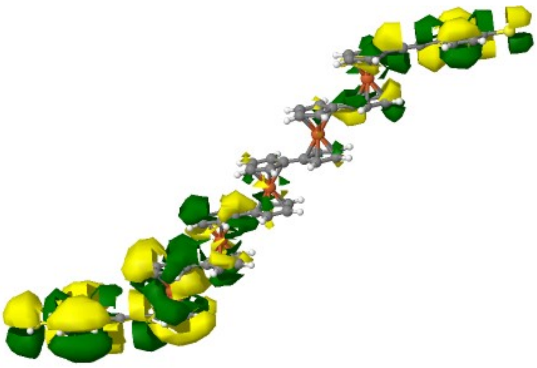

Figure (S6). Frontier molecular orbital landscape of ferrocene derivative **Fe 5**. Computed electronic structures showing the highest occupied molecular orbital (HOMO), lowest unoccupied molecular orbital (LUMO), and their energy levels (HOMO-1 and LUMO+1), illustrating the spatial distribution and energetic relationships of key orbitals involved in charge transport. Here the HOMO and LUMO products are both negative, indicating DQI.

1.3 Binding energy of Au-S

This section presents the binding energy calculations for the five-ferrocene derivatives **Fe 1**–**Fe 5**, focusing on their interaction with Au leads through thiol anchoring groups. All molecules form bonds between the terminal thiol groups and gold electrodes (Au). The density functional theory (DFT) method was employed in conjunction with the counterpoise correction to eliminate the basis set superposition error (BSSE). The binding energy ($B.E$) is computed as the difference between the total energy of the combined system (E_{AB}) and the sum of the energies of the individual components (E_A and E_B), each computed in the presence of ghost atoms [7], [8] [9]:

$$Binding\ Energy = E_{AB} - E_A - E_B$$

Among the five ferrocene units we exhibit identical binding behaviour due to their matching side groups and symmetric structure. The calculated optimised distances and corresponding binding energies are presented in Figure S 7.

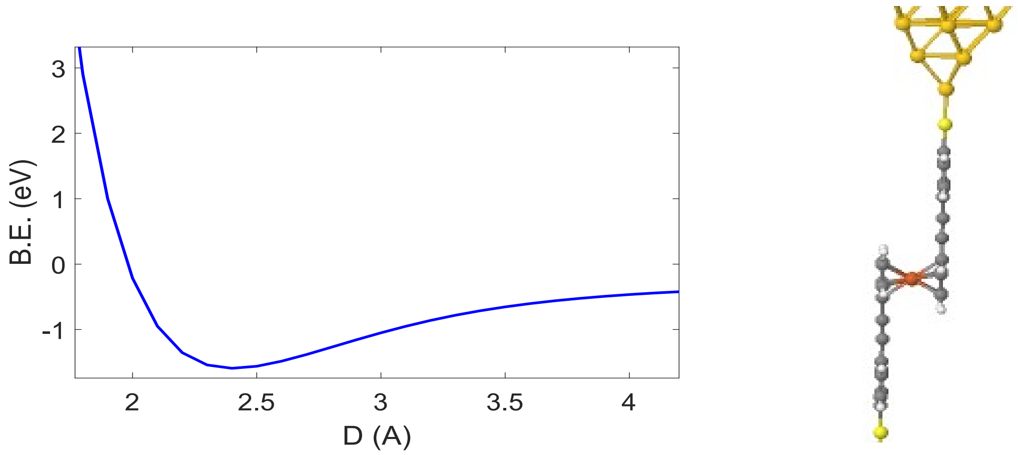

Figure S7: DFT optimisation of ferrocene adsorption on Au(111). (Left) Molecular models illustrating the stable binding configuration of a ferrocene unit functionalized with a thiol anchor group on a gold surface. (Right) The calculated binding energy as a function of the Au-S distance. Geometry optimization reveals a strong chemisorption bond with a binding energy of -1.9 eV at an equilibrium distance of 2.4 Å.

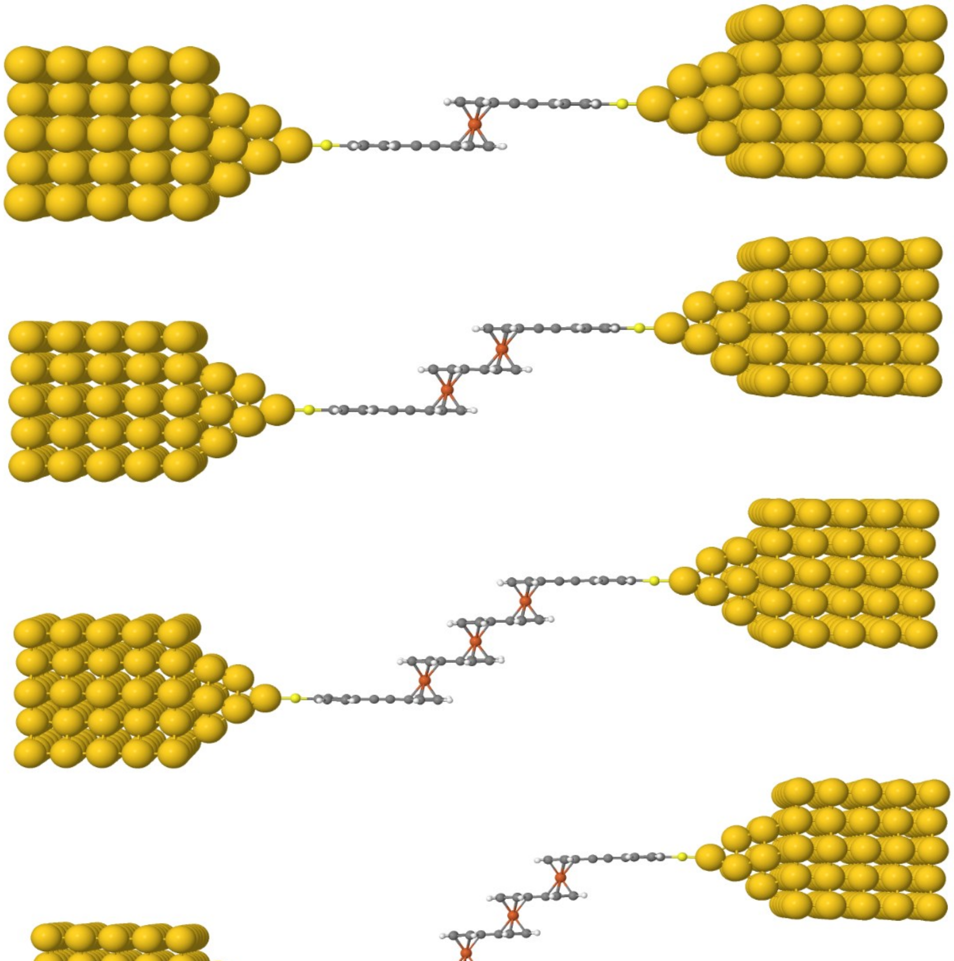

1.4 Transmission coefficient $T(E)$

This section investigates the spin-polarised transmission function of all five ferrocene derivatives (**Fe 1- Fe 5**). The transmission coefficient curves $T(E)$ were obtained using the Gollum quantum transport code, based on the output wavefunction data from DFT calculations. All structures were connected to gold (Au) electrodes through thiol anchoring groups. To ensure proper anchoring geometry, the hydergine atom was removed from the terminal thiol groups [10].

To interpret the transmission characteristics, we classify molecules as HOMO- or LUMO-dominated based on the alignment of the Fermi level relative to molecular orbitals. A HOMO-dominated system has its transmission peak near the HOMO resonance, favouring hole transport, while a LUMO-dominated system would feature transmission near the LUMO level, supporting electron transport.

Our results exhibit HOMO-dominated electronic characteristics based on their transmission spectra as shown in **Figure S 9**. The shared HOMO-dominated nature across all systems suggests that electronic transport is primarily governed by the highest occupied molecular orbital, although the differences in molecular symmetry may contribute to variations in conductance behaviour and interference patterns. incorporates the full electronic structure of the molecules, including orbital interactions, hybridization, and coupling with the electrodes, which makes it more accurate but less transparent. The figure exhibits that the odd ferrocene unit has a destructive quantum interference (DQI), and the even ferrocene units have constructive quantum interference (CQI), and there is no effect of spin on the transmission coefficient.

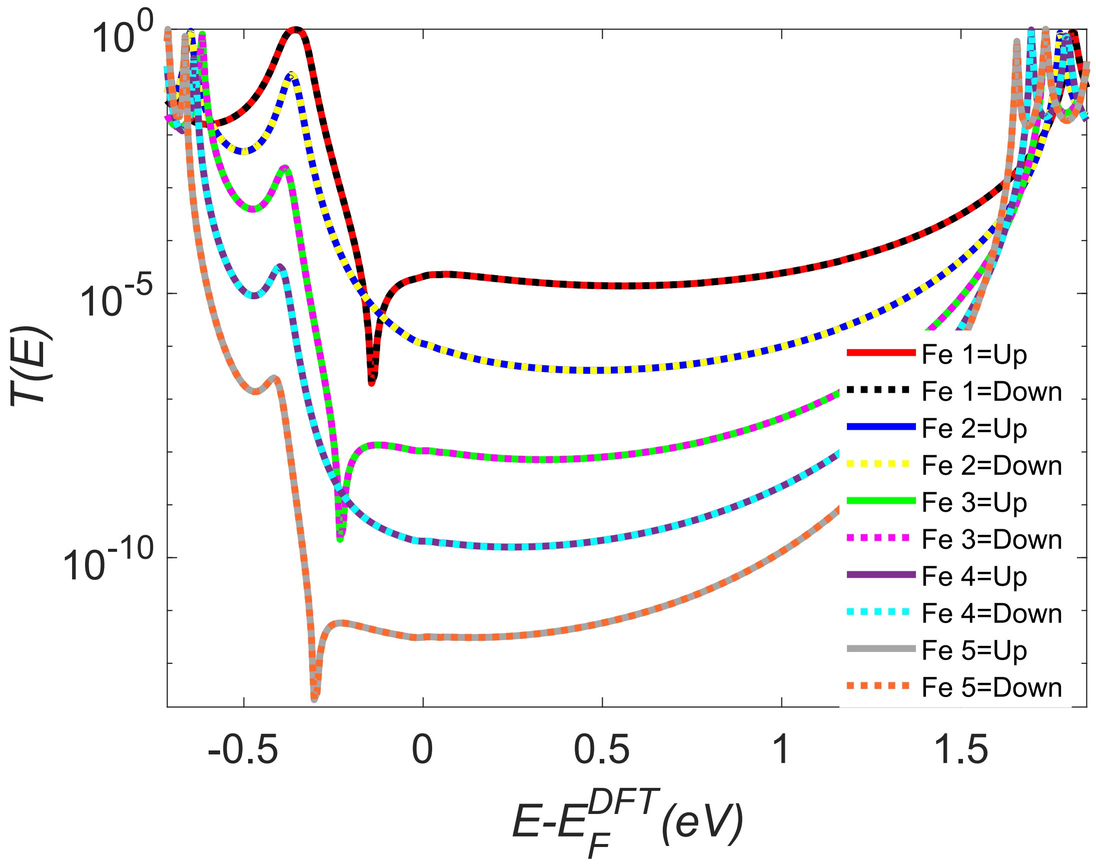

Figure S9: Spin-polarised Transmission coefficients $T(E)$ for all junctions in these studies against the electron energy E.

1.5 Seebeck coefficient

The Seebeck coefficient S, also known as thermopower, measures the voltage generated across a molecular junction in response to a temperature difference between the electrodes. In molecular electronics, S provides valuable information about the type of dominant charge carriers (holes or electrons) and the alignment of frontier orbitals relative to the Fermi energy EF. Within the Landauer formalism, and under the assumption that the transmission function $T(E)$ varies smoothly near E_F, the Seebeck coefficient can be approximated by the Mott formula [11]:

$$S = \frac{-\pi^2 k_B^2 T}{3e} \frac{\partial ln(T(E))}{\partial E}\bigg|_{E=E_F}$$

Here, k_B is Boltzmann's constant, T is temperature, and e is the elementary charge. The sign and magnitude of S depend on the slope of the transmission function near the Fermi level. A positive S implies that hole transport dominates (HOMO-dominated), while a negative S suggests electron transport (LUMO-dominated). The Seebeck profiles shown in **Figure S10** reflect this behaviour, with noticeable variations across all systems in this study due to their differing symmetry and orbital alignment. These findings offer additional confirmation of the HOMO/LUMO nature already observed in the transmission spectra.

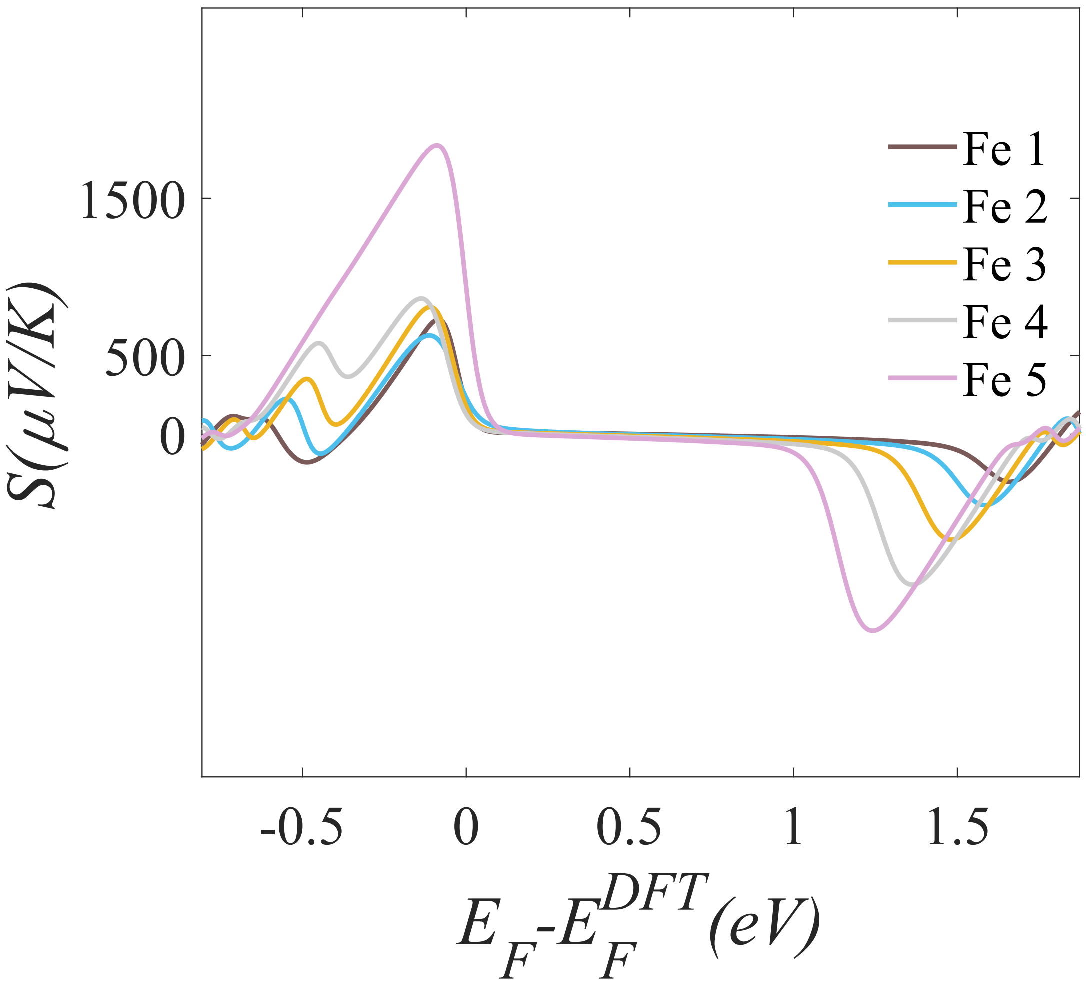

Figure S10: Seebeck coefficient S(E) of ferrocene junctions**.** The Seebeck coefficient as a function of electron energy for all ferrocene units studied.

All molecules exhibit a predominantly positive Seebeck coefficient around the Fermi level, indicating that hole transport dominates. This behaviour aligns with the HOMO-dominated nature of the transmission spectra observed for each molecule, where the Fermi energy lies closer to the highest occupied molecular orbital.

2- Referencing

[1] J. M. Soler *et al.*, 'The SIESTA method for ab initio order-N materials simulation', *J. Phys. Condens. Matter*, vol. 14, no. 11, p. 2745, Mar. 2002, doi: 10.1088/0953-8984/14/11/302.

[2] E. Artacho *et al.*, 'The SIESTA method; developments and applicability', *J. Phys. Condens. Matter*, vol. 20, no. 6, p. 064208, Jan. 2008, doi: 10.1088/0953-8984/20/6/064208.

[3] D. Sánchez-Portal, P. Ordejón, E. Artacho, and J. M. Soler, 'Density-functional method for very large systems with LCAO basis sets', *Int. J. Quantum Chem.*, vol. 65, no. 5, pp.

453–461, 1997, doi: 10.1002/(SICI)1097-461X(1997)65:5<453::AID-QUA9>3.0.CO;2-V.
[4] C. J. Lambert and S.-X. Liu, ‘A Magic Ratio Rule for Beginners: A Chemist’s Guide to Quantum Interference in Molecules’, *Chem. – Eur. J.*, vol. 24, no. 17, pp. 4193–4201, 2018, doi: 10.1002/chem.201704488.
[5] C. J. Lambert and R. Raimondi, ‘Phase-coherent transport in hybrid superconducting nanostructures’, *J. Phys. Condens. Matter*, vol. 10, no. 5, p. 901, Feb. 1998, doi: 10.1088/0953-8984/10/5/003.
[6] Y. Geng *et al.*, ‘Magic Ratios for Connectivity-Driven Electrical Conductance of Graphene-like Molecules’, *J. Am. Chem. Soc.*, vol. 137, no. 13, pp. 4469–4476, Apr. 2015, doi: 10.1021/jacs.5b00335.
[7] N. Kobko and J. J. Dannenberg, ‘Effect of Basis Set Superposition Error (BSSE) upon ab Initio Calculations of Organic Transition States’, *J. Phys. Chem. A*, vol. 105, no. 10, pp. 1944–1950, Mar. 2001, doi: 10.1021/jp001970b.
[8] ‘Counterpoise Correction and Basis Set Superposition Error | PDF | Intermolecular Force | Molecules’, Scribd. Accessed: June 01, 2025. [Online]. Available: https://www.scribd.com/document/421508846/cp
[9] S. F. Boys and F. and Bernardi, ‘The calculation of small molecular interactions by the differences of separate total energies. Some procedures with reduced errors’, *Mol. Phys.*, vol. 19, no. 4, pp. 553–566, Oct. 1970, doi: 10.1080/00268977000101561.
[10] ‘Design and Synthesis of Multipath Compact Cyclophanes for Quantum Interference Studies’, *Eur. J. Org. Chem.*, p. e202400914, Nov. 2024, doi: 10.1002/ejoc.202400914.
[11] C. J. Lambert, ‘Basic concepts of quantum interference and electron transport in single-molecule electronics’, *Chem. Soc. Rev.*, vol. 44, no. 4, pp. 875–888, 2015, doi: 10.1039/C4CS00203B.
